# Changing Oar Rotation Axis Position Increases Catch Angle During Indoor and In-Field Para-Rowing: A Randomized Crossover Trial Verified by a Repeated Measurement Trial

**DOI:** 10.3389/fphys.2022.833646

**Published:** 2022-02-22

**Authors:** Steffen Held, Ludwig Rappelt, Pamela Wicker, Lars Donath

**Affiliations:** ^1^Department of Intervention Research in Exercise Training, German Sport University Cologne, Cologne, Germany; ^2^Department of Sports Science, Bielefeld University, Bielefeld, Germany

**Keywords:** gearing, multiple testing, Paralympic, single case, biomechanics, spinal cord injury

## Abstract

A long rowing stroke length is crucial for adequate rowing performance. Therefore, the relocation of the oar from traditional “in front” (NORM) to “behind the rotation axis” (GATE) may increase (para) rowing performance. Thus, 15 able-bodied rowers (21.4 ± 3.6 years; 187 ± 8 cm; 85.4 ± 8.2 kg) completed indoor TANK rowing 2 min TimeTrials (2 min-TT) of GATE and NORM in a randomized order. Additionally, one elite Paralympic oarsman (37 years, 185 cm, 67 kg) performed a multiple single case in-field BOAT testing (24x2min-TT of GATE and NORM in a randomized order). GATE revealed significantly larger catch angles during TANK (+97.1 ± 120.4%; *p* = 0.001, SMD = 0.84) and BOAT (+11.9 ± 3.2%; *p* < 0.021; SMD = 2.69; Tau-U = 0.70) compared to NORM. While total stroke length, rowing power, and work per stroke increased in GATE during TANK (*p* < 0.010, SMD > 0.634), no such significant changes of these performance parameters between GATE and NORM were observed during BOAT (*p* > 0.021; SMD < 0.58; Tau-U < 0.29). Rowing economy-related parameters (power or speed per oxygen uptake) and boat speed also showed no significant differences between GATE und NORM during BOAT (*p* > 0.61; SMD < 0.31; Tau-U < 0.19). The shape of the force–angle curve (position of peak force and ratio between average and maximal force) remained unaffected from GATE during both TANK (*p* > 0.73, SMD < 0.1) and BOAT (*p* > 0.63; SMD < 0.60; Tau-U < 0.27). In conclusion, GATE shifted the entire rowing stroke towards the catch (+6.6 ± 1.8°) without notably affecting relevant performance parameters during BOAT. Particularly during crew rowing, the minimization of detrimental boat movements for perfect synchrony should be aimed for. Accordingly, the combined application of GATE and NORM (for different athletes in crew boats) may be beneficial for rowing synchronization.

## Introduction

Para-rowing is a competitive and recreational sportive activity that gained growing worldwide popularity (Lewis, 2011). Para-rowing was firstly introduced to the Paralympics at the 2008 Beijing Games. Para-rowing programs allow and encourage sports participation by individuals with functional, intellectual, or visual disabilities. According to the elite-standard para-rowing classification regulations of the World Rowing Association, three categories of para-rowing exist (FISA, 2017): Legs, trunk, and arms (LTA), trunk and arms (TA), and arms and shoulders (AS) rowing. LTA rowing is similar to able-bodied rowing using a sliding seat and having no movement restrictions. The AS category (e.g., *PR1 Single Sculls*) of para-rowing is characterized by motions of the AS for propulsion (FISA, 2017). Athletes within this category have minimal or no trunk and leg function and thus require additional stability by a fixed seat back, to which the torso of the AS rower is strapped across the thoracic region (Cutler et al., 2017). Due to these restrictions, the rowing stroke length of TA and AS rowing is significantly shorter compared to conventional LTA rowing (Cutler et al., 2017). A long rowing stroke length, however, is crucial for adequate rowing performance (Baudouin and Hawkins, 2004; Kleshnev, 2016). In this regard, large oar angles in the frontal reversal of the rowing motion (catch) have been shown to increase propulsion through enhanced utilization of the hydrodynamic lift (Baudouin and Hawkins, 2004).

**Figure 1:**
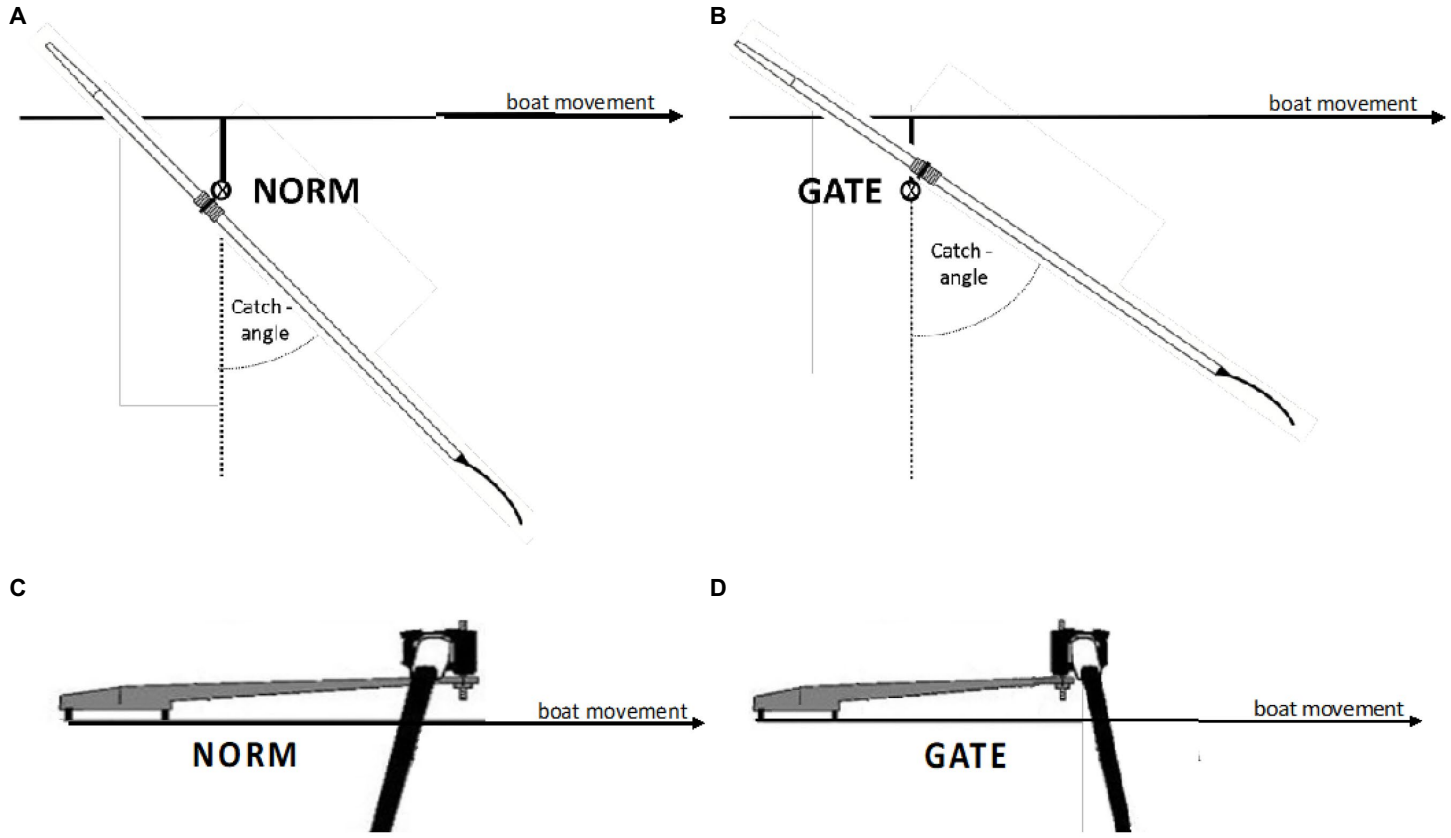
Schematic representation of the standard setup (**A,C**; NORM) and the modified version (**B,D**; GATE).

The stroke length can be modified by moving the rotation axis of the oars (in relation to the boat; Dudhia, 2007; Kleshnev, 2016). A displacement of the rotation axis inwards (toward the center of the boat) generates an increased catch angle (Kleshnev, 2016). Accordingly, the displacement of the oar from in front of the rotation axis (NORM) to behind of the rotation axis (GATE) may result in larger catch angles (Figure 1). Subsequently, these larger catch angles could produce longer overall stroke length, which may increase the resulting rowing power.

Against this background, the present study aimed to analyze these hypotheses. By employing biomechanical measurements during indoor tank (Trompeter et al., 2019) and in-field *PR1 single scull* (FISA, 2017) rowing, we examined whether GATE vs. NORM resulted in higher rowing power and boat speeds. Since access to participants in competitive sports, and especially in para-sports, is heavily limited, an alternative research setup was chosen. First, a proof-of-concept trial was completed with able-bodied participants (indoor rowing tank measurements), in order to test, whether the displacement of the oar from in front of the rotation axis to behind results in larger catch angles. Subsequently, these results were re-examined in a repeated single case study design with multiple testing (Parker et al., 2011; Lee and Cherney, 2018) of one elite Paralympic oarsman (in-field boat measurements). Thereby, the proof-of-concept data were used for power estimation of the repeated single case measurements. Our findings might deliver new insights into the biomechanical setup of para-rowing and biomechanical optimization of para-sport setups.

## Materials and Methods

### Proof-of-Concept Testing—Indoor Rowing Tank Measurements

#### Participants

In accordance with previous para-rowing studies (Cutler et al., 2017), able-bodied athletes were enrolled in a para-rowing setup (described in the following section). Assuming large effect sizes (*η_p_*^2^ = 0.14; *f* = 0.41) and high correlations (*r* = 0.70) between measurements (Cohen, 1988), a previously conducted power analysis (*α* = 0.05, study power (1-β-error) = 0.95, g*Power, Version 3.1.9.6) revealed a sample size of *n* = 15. Therefore, 15 experienced male rowers (21.4 ± 3.6 years; 187 ± 8 cm; 85.4 ± 8.2 kg, >5 years of rowing experience) volunteered in this proof-of-concept testing. Inclusion criteria were (I) at least 18 years of age and (II) no health complaints and other disease conditions. Before testing, all participants refrained from any strenuous exercise for 48 h. All participants of the proof-of-concept testing and single case repeated measurements signed an informed consent after receiving relevant study information. The proof-of-concept testing and single case repeated measurements study protocol complied with the Declaration of Helsinki, has been approved by the ethics committee of the German Sport University Cologne (172/2018), and fulfilled the international ethical standards (Harriss and Atkinson, 2015).

#### Design

The proof-of-concept testing part was conducted as a randomized controlled crossover trial employing an indoor rowing tank. The indoor rowing tank is considered a well-established indoor testing approach that properly simulates an open-water situation (Trompeter et al., 2019). Thereby, rowers sit in a fixed rowing position with 2 (scull) oars, surrounded by a channel of water (Trompeter et al., 2019). After a standardized 15-min warm up (rowing at low intensity/heart rate, which corresponds to a blood lactate concentration < 2 mmol/L), two 2-min time trials (5 min rest in between) were completed in a randomized order, with a standard oarlock (Figures 1A,C; NORM; *Concept 2*, Morrisville, United States) and a modified oarlock [Figures 1B,D; GATE; *Institut für Forschung und Entwicklung von Sportgeräten* (FES), Berlin, Germany]. Both 2-min time trials were performed at maximum intensity with a fixed stroke rate of 34 spm. The last min of each 2-min time trial was included for further analysis. To simulate the (no leg) rowing motion of para-athletes, the legs and trunk were fixed with straps. A familiarization session (20 min tank rowing at low intensity/heart rate, which corresponds to a blood lactate concentration < 2 mmol/L and several short burst at high intensity) was completed 1 week prior to testing.

#### Data Collection

A radio telemetry system (*BioRowTel System*, Biorow, Berkshire, Great Britain) was used for data acquisition (25 Hz sampling frequency) during tank rowing. The force applied to the oar-handle was measured using a strain gauged transducer attached to the oar shaft (*BioRowTel System*, Biorow, Berkshire, Great Britain, accuracy ± 0.5%). Each oar was dynamically calibrated before each session using a precision load cell. The oar angles in horizontal and vertical dimensions were measured using conductive plastic potentiometers (*BioRowTel System*, Biorow, Berkshire, Great Britain, accuracy ±0.1%). Total rowing angle (angle; ±0.1%), catch angle (±0.1%), stroke rate (rate; ±0.1%), rowing power (P_row_; ±0.5%), and work per stroke (WPS; ±0.5%) were determined (Kleshnev, 2016; Held et al., 2019) accordingly. To quantify the shape of the force–displacement curve, the position of peak force (Peak_Position_; ±0.1%) and the ratio between average and maximal force (Ratio_mean−max_) were determined (Kleshnev, 2016).

#### Statistics

Data are presented as mean ± SD. After verifying normal distribution and variance homogeneity, pairwise comparisons (pairwise *t*-test) between NORM and GATE were performed for each output parameter (P_row_, WPS, total angle, catch angle, stroke rate, Peak_Position_, and Ratio_mean−max_). For pairwise effect size comparison, standard mean differences (SMD) were additionally calculated as between mode differences divided by the pooled standard deviations of both modes (trivial: SMD < |0.2|, small: |0.2| ≤ SMD < |0.5|, moderate: |0.5| ≤ SMD < |0.8|, large SMD ≥ |0.8|; Cohen, 1988).

### Single Case Repeated Measurements—In-Field Boat Measurements

#### Participant

One elite Paralympic oarsman (37 years, 185 cm, 67 kg; 8 years Olympic rowing experience) was tested in a single case (n-of-1) design using repeated in-field rowing (boat) measurements. This elite athlete took part in several Paralympic, World, and European championships. Due to a clinically motor complete spinal cord injury (T6), this athlete was classified in the AS classification.

#### Design

A multiple (repeated) testing design was then used for the (n-of-1) in-field boat trial. Therefore, the data obtained from the indoor rowing tank were used for the sample size estimation of this in-field boat measurements. Based on indoor rowing tank measurements (P_row_; Table 1), an estimation of the necessary measurement repetitions (*α* = 0.10; study power (1-β-error) = 0.95; effect size SMD = 0.855; no baseline drift) required a number of samples/measurements of *n* = 21 (Percha et al., 2019). In conclusion, in-field boat measurements in the *PR1 single scull* mode were repeated 24 times (12× GATE; 12× NORM). These 24 measurement trials were spread over six testing days. Between each of the six testing days, 1 week of training (alternating between GATE and NORM, for 5 sessions) was conducted. On each testing day, four 2-min time trials (with 5 min rest in between) were completed in randomized order, two with GATE and two with NORM. Both 2-min time trials were performed at maximum intensity with a fixed stroke rate of 34 spm. The last minute of each 2-min time trial was included into further analysis. Similar to the indoor tank measurements, a standardized 10-min warm up (as described above) was performed prior to each in-field measurement. In order to control the circadian effects on performance, all measurements were conducted at similar times of day. All in-field measurements were carried out in the participant’s own (accustomed) *PR1 single scull* para-boat.

**Table 1:** Output parameter of the proof-of-concept testing (indoor rowing tank measurements).

	NORM	GATE	*p*	SMD
Stroke rate (spm)	34.3 ± 2.8	34.2 ± 3.4	0.980	−0.032
P_row_ (W)	101 ± 44	146 ± 60	0.006	0.855
WPS (J)	177 ± 82	261 ± 121	0.010	0.813
Angle (°)	44.8 ± 9.4	52.4 ± 14.1	0.010	0.634
Catch angle (°)	9.4 ± 7.3	18.0 ± 12.5	0.001	0.840
Peak_Position_ (%)	39.5 ± 5.6	40.1 ± 7.2	0.730	0.093
Ratio_mean−max_ (%)	48.2 ± 5.0	48.1 ± 4.9	0.914	−0.020

P_row_: rowing power; WPS: work per stroke; Angle: total rowing angle; Catch angle; Peak_Position_: position of peak force; Ratio_mean−max_: ratio between average and maximal force. In addition, the Bonferroni t-test results (*p* value) and pairwise effect sizes (SMD) are displayed.

#### Data Collection

In addition to the indoor rowing measurement setup, the average boat speed (v_boat_) was determined by a 10 Hz-GPS (*BioRowTel Systems*, Biorow, Berkshire, Great Britain, accuracy ± 0.1 m s^−1^) during in-field boat measurements. Furthermore, oxygen uptake (VO_2_) data were collected with a breath-by-breath spiroergometric system (*Metamax 3b*, Cortex Biophysics, Leipzig, Germany) during field rowing. The technical error of measurement of the device is reported to be less than 2% (Macfarlane and Wong, 2012). The spiroergometric system was calibrated prior to each test following the manufacturer’s recommendations. Objective exhaustion were verified for each in-field boat measurement trials following the criteria by Midgley et al. (2007). In-field rowing efficiency was determined as rowing power per oxygen uptake (P_VO2_) and boat speed per oxygen uptake (v_VO2_).

#### Statistics

All single case repeated measurement data are presented as mean ± SD. Normal distribution of all output variables was verified using Shapiro–Wilk tests, and variance homogeneity was visually verified *via* plotting residuals. Repeated measures analysis of variances (rANOVA) were conducted to examine “mode” differences (GATE vs. NORM) for the respective outcome measures (P_row_, WPS, total angle, catch angle, stroke rate, Peak_Position_ and Ratio_mean−max_, P_VO2_, and V_VO2_) during in-field boat measurements. Effect sizes for rANOVA were given as partial eta squared (*η_p_*^2^), with values ≥0.01, ≥ 0.06, and ≥ 0.14 indicating small, moderate, and large effect sizes, respectively (Cohen, 1988). In case of significant rANOVA effects, Bonferroni *post-hoc* tests were subsequently computed. Further, for pairwise effect size comparison, SMD were calculated (Cohen, 1988). The multiple testing outcome measures of the in-field boat measurements were visually analyzed using percentage of non-overlapping data (PND), percentage exceeding the median (PEM), percentage exceeding the trend (PET), non-overlap of all pairs (NAP), percentage all non-overlapping data (PAND), mean difference between both conditions (MD), trend difference 400 between both conditions (Δtrend), and SMD (Horner et al., 2005; Kratochwill et al., 2010). Furthermore, Tau-U effect sizes (Parker et al., 2011; Lee and Cherney, 2018) were calculated to complement visual analysis using the “scan” package for R (Wilbert and Lueke, 2021). Tau-U is a non-parametric effect size analysis that examines non-overlap between phases (Parker et al., 2011; Lee and Cherney, 2018) and corrects for undesirable baseline trends (Vannest and Ninci, 2015). Tau-U effect size scores ≥0.01, ≥ 0.20, ≥ 0.60, and ≥ 0.80 indicate small, moderate, large effects, and very large effects, respectively (Vannest and Ninci, 2015). All statistical analyses (for the proof-of-concept testing and single case repeated measurements) were conducted using R (version 4.0.5) and RStudio (version 1.4.1106) software.

## Results

### Indoor Rowing Tank Measurements

**Figure 2:**
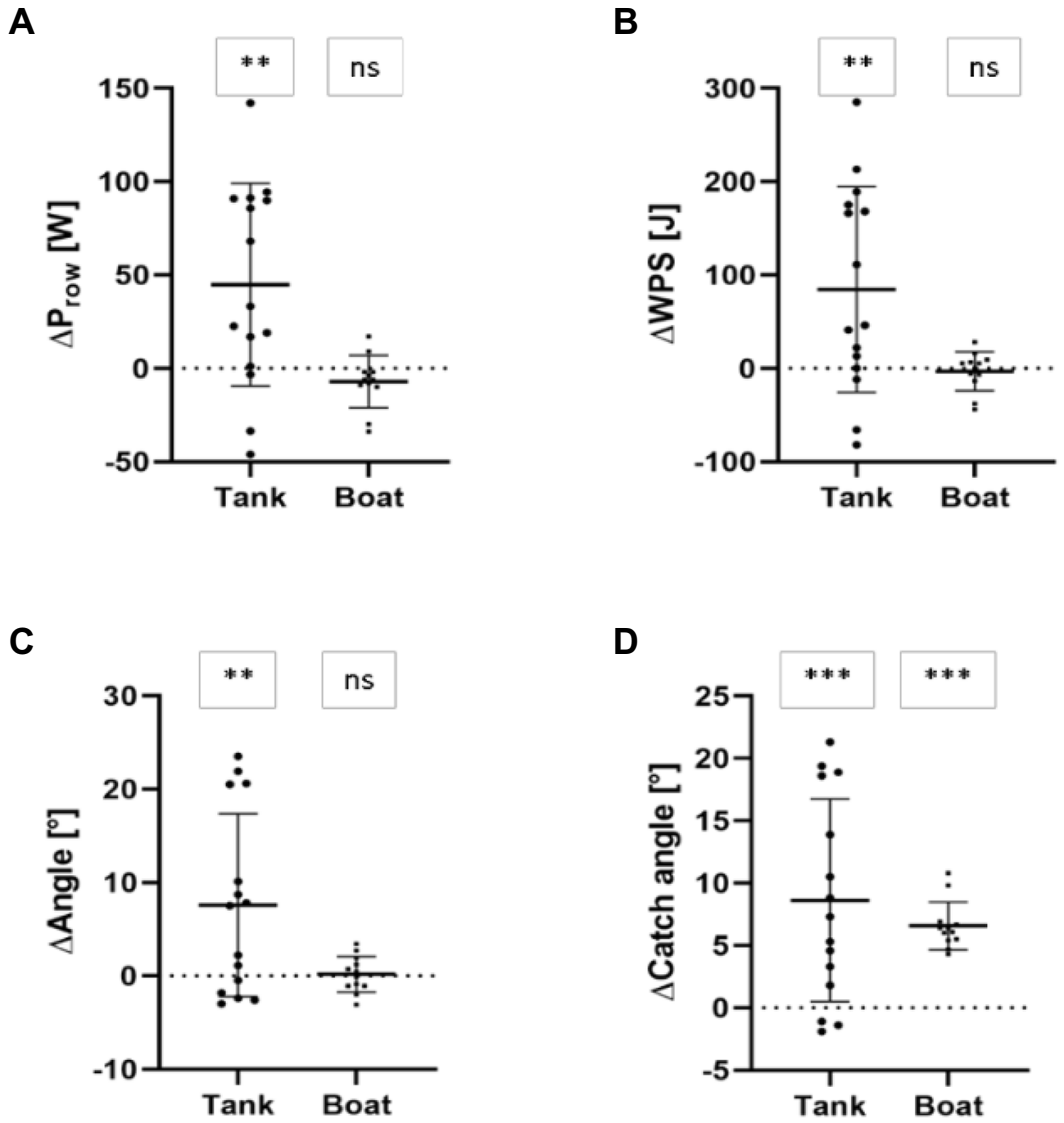
Change scores of rowing power (ΔP_row_; **A**), work per stroke (ΔWPS; **B**), total rowing angle (ΔAngle; **C**) and catch angle (ΔCatch angle; **D**) during indoor rowing tank and in-field boat measurements. Note: ^***^p < 0.001; ^**^p < 0.01; ns = not significant (*p* > 0.05).

**Figure 3:**
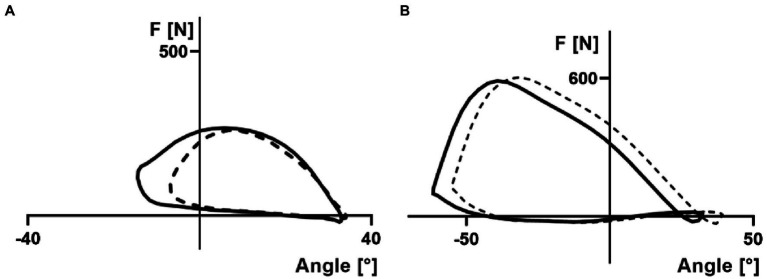
Representation of the average handle force (F) as a function of rowing angle during proof-of-concept (**A**; indoor rowing tank measurements) and single case repeated measurement (**B**; in-field boat measurements) and indoor rowing tank measurements **(B)**. GATE is displayed in solid lines and NORM in dashed lines.

Stroke rate and shape of force–angle curve (Peak_Position_, Ratio_mean−max_) revealed no significant differences (*p* ≥ 0.730; SMD ≤ 0.09; Table 1) during indoor tank rowing measurements. In contrast, rowing power (P_row_ + 55.8 ± 57.3%; Figure 2A), work per stroke (WPS +59.7 ± 67.2%; Figure 2B), total angle (+19.9 ± 23.9; Figures 2C, 3A), and catch angle (+97.1 ± 120.4%; Figures 2D, 3B) increased significantly (*p* ≤ 0.010; SMD ≥ 0.63; Table 1) from NORM to GATE, during indoor tank rowing.

### In-Field Boat Measurements

During in-field measurements (Table 1) the rANOVA revealed no statistically significant mode × time interactions (*p* ≥ 0.066; *η_p_*^2^ ≤ 0.60; Table 2) for all output parameters [rate, v_boat_, P_row_ (Figure 2A), WPS (Figure 2B), Angle (Figure 2C), shape of force–angle curve (Peak_Position_, Ratio_mean−max_), power per oxygen uptake (P_VO2_), and boat speed per oxygen uptake (V_VO2_)] except of catch angle (*p* ≤ 0.001; *η_p_*^2^ = 0.81; Table 2). *Post-hoc* tests revealed statistically significant (*p* = 0.021; SMD = 2.70) larger catch angle (+11.9 ± 3.2%) for GATE compared to NORM during all in-field measurement trials (Figure 2C).

**Table 2:** Output parameter and overlapping indices of the single case repeated measurements (in-field rowing measurements).

	Stroke rate (spm)	v_boat_ (m s^−1^)	P_row_ (W)	WPS (J)	Angle (°)	Catch angle (°)	Peak_Position_ (%)	Ratio_mean−max_ (%)	P_VO2_ (W min^−1^ L^−1^)	v_VO2_ (m s^−1^ min^−1^ L^−1^)
NORM	33.9 ± 1.0	3.23 ± 0.21	303 ± 13	537 ± 21	94.3 ± 1.7	55.2 ± 1.9	24.9 ± 1.9	51.3 ± 1.8	117 ± 9	1.26 ± 0.14
GATE	33.3 ± 1.1	3.17 ± 0.17	296 ± 11	534 ± 21	94.5 ± 1.8	61.8 ± 2.9	23.9 ± 0.9	52.2 ± 1.9	116 ± 9	1.24 ± 0.12
*p*	0.955	0.997	0.999	0.990	0.066	0.001	0.063	0.134	0.614	0.786
*η_p_* ^2^	0.091	0.028	0.017	0.047	0.599	0.810	0.603	0.525	0.269	0.192
SMD	−0.571	−0.314	−0.581	−0.143	0.114	2.692	−0.673	0.486	−0.111	−0.160
PND	0.00	8.33	0.00	8.33	8.33	83.33	0.00	8.33	8.33	8.33
PEM	25.00	16.67	33.33	41.67	50.00	100.00	25.00	75.00	33.33	41.67
PET	0.00	75.00	41.67	83.33	75.00	100.00	66.67	25.00	58.33	75.00
NAP	30.56	36.81	34.38	47.22	48.96	98.61	31.25	66.67	44.44	47.92
PAND	25.00	37.50	37.50	41.67	50.00	91.67	33.33	66.67	41.67	50.00
Tau-U	−0.29	−0.19	−0.23	−0.04	−0.02	0.70	−0.27	0.24	−0.08	−0.03
MD	−0.59	−0.06	−7.08	−3.02	0.17	6.58	−0.01	0.01	−1.04	−0.01
Δtrend	0.05	0.02	0.49	−0.06	−0.06	0.19	0.00	0.00	1.46	0.02
SMD	−0.58	−0.29	−0.51	−0.14	0.09	3.40	−0.51	0.50	−0.11	−0.05

Rate: stroke rate; v_boat_: boat speed; P_row_: rowing power; WPS: work per stroke; Angle: total rowing angle; Catch angle; Peak_Position_: position of peak force; Ratio_mean−max_: ratio between average and maximal force; P_VO2_: rowing power per oxygen uptake; v_VO2_: boat speed per oxygen uptake; mode × time rANOVA interaction (*p*) and effect size (*η_p_*^2^) are displayed separately for each rowing condition; PND: percentage of non-overlapping data; PEM: percentage exceeding the median; PET: percentage exceeding the trend; NAP: non-overlap of all pairs; PAND: percentage all non-overlapping data; Tau-U: Tau-U effect size; MD: mean difference between both conditions; Δtrend: trend difference between both conditions; SMD: standardized mean difference.

**Figure 4:**
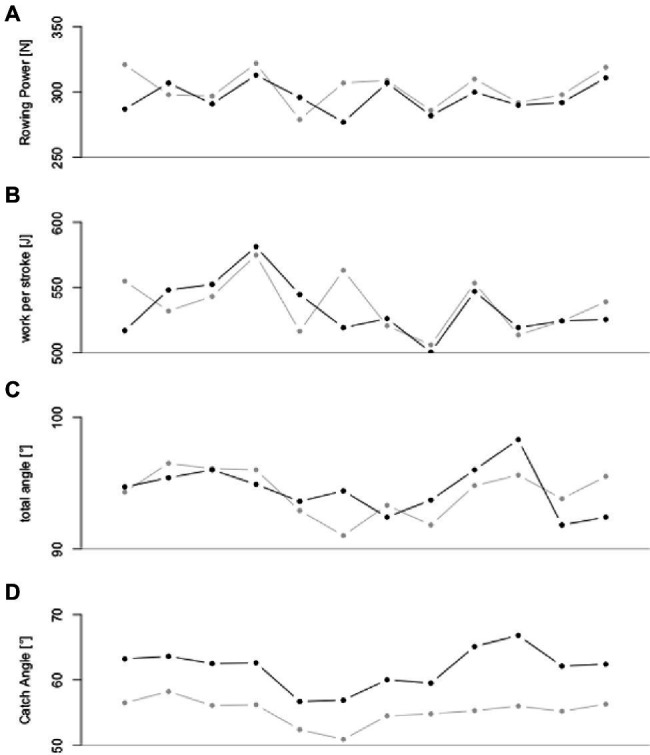
Rowing power **(A)**, work per stroke **(B)**, total angle **(C)**, and catch angle **(D)** data (means) for both NORM (grey) and GATE (black) during in-field boat measurement of the elite para-rower.

Percentage of non-overlapping data (PND), percentage exceeding the median (PEM), percentage exceeding the trend (PET), non-overlap of all pairs (NAP), and percentage all non-overlapping data (PAND) ranged between 0 and 83.3% (Table 2) for stroke rate, boat speed (v_boat_), rowing power (P_row_; Figure 4A), work per stroke (WPS, Figure 4B), total angle (Figure 4C), Peak_Position_, Ratio_mean−max_, and rowing economy (P_VO2_: power per oxygen uptake; V_VO2_: boat speed per oxygen uptake). In addition, rate, v_boat_, P_row_, WPS, Angle, Peak_Position_, Ratio_mean−max_, P_VO2_, and V_VO2_ showed only trivial to moderate SMD and small to moderate Tau-U effect sizes (Table 2). In contrast, catch angle (Figure 4D) revealed percentage of non-overlapping data (PND), percentage exceeding the median (PEM), percentage exceeding the trend (PET), non-overlap of all pairs (NAP), and percentage all non-overlapping data (PAND) ≥ 83.3% (Table 2). In addition, large SMD and Tau-U effect sizes revealed increased catch angles (Table 2) during GATE compared to NORM during in-field boat measurements.

## Discussion

To the best of our knowledge, this is the first study that comparatively examined different (para) boat setups during indoor tank rowing and in-field rowing including a single subject approach. We first conducted a randomized crossover testing as an initial proof-of-concept study with able-bodied participants (indoor rowing tank measurements) with a subsequent verification *via* multiple single case testing of an elite Paralympic oarsman (in-field boat measurements). Our key findings indicate that the displacement of the oar from in front of the rotation axis (NORM) to behind the rotation axis (GATE) resulted in significantly larger catch angles during both indoor tank and in-field *PR1 single scull* rowing. While total stroke length, rowing power, and work per stroke increased in GATE during indoor tank rowing, no such significant changes of these performance parameters between GATE and NORM were observed during the in-field boat condition. Rowing economy-related parameters (power or speed per oxygen uptake) and boat speed also showed no significant differences between GATE und NORM during in-field rowing. Interestingly, the shape of the force–angle curve (position of peak force and ratio between average and maximal force) remained unaffected from GATE during both indoor rowing tank and in-field boat measurements.

The observed rowing angle changes after modifying the oar-boat setups are in line with previous scientific research findings in rowing (Kleshnev, 2016; Held et al., 2019). In contrast to the current study, however, previous rowing angle changes were merely induced by changes of the gearing ratio between inner and outer lever of the oar (Held et al., 2019). Our study was the first that showed the effect of the displacement of the rotation axis on the rowing angle. The results indicate that GATE (compared to NORM) enables larger catch angles during both indoor tank and in-field rowing, but larger total stroke lengths were only observed during indoor tank rowing. Based on enhanced utilization of the hydrodynamic lift (Baudouin and Hawkins, 2004), larger catch angles are considered favorable for the rowing performance (Baudouin and Hawkins, 2004; Kleshnev, 2016). The observed differences between indoor tank and in-field rowing might result from the following factors: By fixing the legs and hips during indoor tank rowing, the able-bodied athletes could use an arm, shoulder, and despite fixation partly trunk movement for propulsion. In contrast, the *PR1 single scull* para-athlete could only use the arms and shoulders for propulsion. Correspondingly, the indoor tank rowing setup may allow a large increments of stroke length. In addition, according to the elite-standard Para-Rowing Classification Regulations of the World Rowing Association, the used indoor rowing tank setup seems to be more suitable as trunk and arms (TA) and the *PR1 Single Scull* of the para-athlete as arms and shoulders (AS) rowing (FISA, 2017).

The shape of the force–angle curve (Kleshnev, 2016) was characterized by the position of the peak (in relation to the stroke length; Peak_Position_) and the ratio of peak force to average force (Ratio_mean−max_). GATE showed no effect on these two parameters (Peak_Position_ and Ratio_mean−max_) for both indoor tank and in-field rowing. Accordingly, the in-field rowing results showed that GATE shifted the entire rowing stroke by 6.6 ± 1.8° toward the catch position without affecting other parameters like total stroke length, rowing power, work per stroke, rowing economy (power or boat speed per oxygen uptake) and shape of force–angle curve. In general, the goal in competitive rowing is to cover a 2000 m race distance in the shortest amount of time. Accordingly, each rower or rowing crew aims at maximizing power output and minimizing power losses to achieve maximum average boat velocity. During crew rowing, it is the collective performance of the crew that affects the movements of the boat (Wing and Woodburn, 1995; Hill, 2002; Baudouin and Hawkins, 2004). Particularly for crew rowing, the coaching literature (O’Brien, 2011) and also scientific research (Wing and Woodburn, 1995; Baudouin and Hawkins, 2004; Cuijpers et al., 2017) suggest to minimize detrimental boat movements for perfect synchrony. Improved synchrony in the crew boat can be achieved by adjusting the rowing angles (total stroke length and catch angle) of each oarsman. Accordingly, our results enable to increase the crew boat synchronicity by matching the catch angles using GATE or NORM for different athletes in crew boats. Therefore, the combination of GATE and NORM enables a broader range of rowing angles enabling a better exploration for synchronized kinematics of the entire rowing crew, aiming at minimizing detrimental boat movements. For this purpose, GATE and NORM were already used in a crew boat at the 2019 World Rowing Championships.

Apart from the able-bodied participants, only one elite Paralympic oarsman was examined. However, the number of elite athletes and para-athletes in particular is very limited. Nevertheless, the combination of proof-of-concept (indoor rowing tank) and multiple single case (in-field boat) testing allows statistically relevant conclusions despite the small sample size. Furthermore, the results show that findings from able-bodied athletes measured in a para-setup are only partially transferable to the para-athlete. Therefore, the chosen research design (randomized crossover testing as proof-of-concept and subsequent verification *via* multiple single case testing) can also serve as a template for further research in the para-sport field.

In conclusion, the displacement of the oar from in front of the rotation axis (NORM) to behind the rotation axis (GATE) resulted in significant and meaningful larger catch angles during indoor tank and in-field *PR1 single scull* rowing. While total stroke length, rowing power and work per stroke increased in GATE only during tank rowing, no meaningful changes of these performance parameters between GATE and NORM were observable during in-field boat measurements. Therefore, GATE shifted the entire rowing stroke toward the catch position without affecting other parameters, such as total stroke length, rowing power, work per stroke, rowing economy (power or boat speed per oxygen uptake), and shape of force–angle curve. Since synchrony in the crew boat can be improved by adjusting the rowing angles of each crew member, the combination of GATE and NORM in crew boats seems reasonable to enhance synchronization which is essential for optimal propulsion.

## Data Availability Statement

The raw data supporting the conclusions of this article will be made available by the authors, without undue reservation.

## Ethics Statement

The studies involving human participants were reviewed and approved by Ethics committee, German Sport University Cologne. The patients/participants provided their written informed consent to participate in this study.

## Author Contributions

SH, LR, and LD contributed to the conception and design of the study. SH led the intervention and wrote the first draft of the manuscript. SH and LR performed the statistical analysis. SH, LR, PW, and LD wrote sections of the manuscript. PW copyedited the draft for content, language, and format and organized the submission and revision/resubmission process. All authors contributed to the article and approved the submitted version.

## Funding

This work was financially supported by the German Federal Institute of Sports Sciences (BISp) under Grant (ZMVI4-072031/18-19). In addition, we acknowledge the financial support of the German Research Foundation (DFG) and the Open Access Publication Fund of Bielefeld University for the article processing charge.

## Conflict of Interest

The authors declare that the research was conducted in the absence of any commercial or financial relationships that could be construed as a potential conflict of interest.

## Publisher’s Note

All claims expressed in this article are solely those of the authors and do not necessarily represent those of their affiliated organizations, or those of the publisher, the editors and the reviewers. Any product that may be evaluated in this article, or claim that may be made by its manufacturer, is not guaranteed or endorsed by the publisher.
